# Diolistics: incorporating fluorescent dyes into biological samples using a gene gun

**DOI:** 10.1016/j.tibtech.2007.07.014

**Published:** 2007-11

**Authors:** John A. O’Brien, Sarah C.R. Lummis

**Affiliations:** 1 Neurobiology Division, MRC Laboratory of Molecular Biology, Hills Road, Cambridge, UK, CB2 2QH; 2 Dept of Biochemistry, University of Cambridge, Cambridge UK, CB2 1QW

## Abstract

The hand-held gene gun provides a rapid and efficient method of incorporating fluorescent dyes into cells, a technique that is becoming known as diolistics. Transporting fluorescent dyes into cells has, in the past, used predominantly injection or chemical methods. The use of the gene gun, combined with the new generation of fluorescent dyes, circumvents some of the problems of using these methods and also enables the study of cells that have proved difficult traditionally to transfect (e.g. those deep in tissues and/or terminally differentiated); in addition, the use of ion- or metabolite-sensitive dyes provides a route to study cellular mechanisms. Diolistics is also ideal for loading cells with optical nanosensors – nanometre-sized sensors linked to fluorescent probes. Here, we discuss the theoretical considerations of using diolistics, the advantages compared with other methods of inserting dyes into cells and the current uses of the technique, with particular consideration of nanosensors.

## Introduction

The ability to visualize cells using differential labelling techniques has had a central role in biology since the beginning of the 20th century. The classic example is the Golgi stain, developed by Camillo Golgi, and elevated to an art form by Ramon y Cajal. Labels, such as the Golgi stain, however, which enable us to visualize the intricate structural details of individual cells, are time consuming and can only be used on post-mortem tissue, thereby limiting their value for many physiological studies. The new generation of fluorescent dyes, which can be visualized within minutes after incorporation into living tissue using the gene gun, have revolutionised our ability to examine cells and cellular processes.

**Figure 1:**
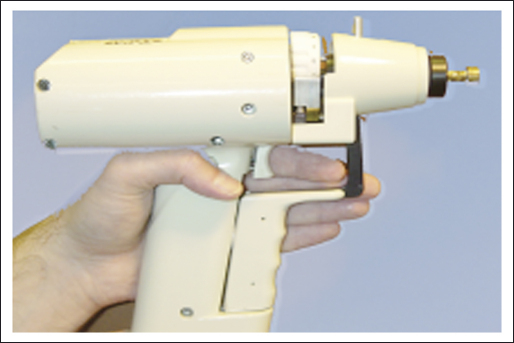
A gene gun. The Helios (Bio-Rad) gene gun is a small portable instrument that enables efficient transportation of dyes into cells – a technique that is becoming known as diolistics.

The gene gun was developed initially by Sanford, Klein and Wolfe at Cornell University as a mechanism of introducing new genetic material into plant cells [1]. The original gene gun transfection procedure involved placing the sample in a sealed chamber, which was then placed under vacuum before firing DNA-coated microparticles into the preparation. This procedure severely limited the applications for which the gene gun could be used because samples needed both to be relatively small and resistant to damage when placed in a vacuum. The gene guns of today are more sophisticated and, in particular, do not require the use of an evacuated chamber. The more recent versions, such as the BioRad (Helios™; http://www.bio-rad.com/) hand-held gene gun (Figure 1), as well as several custom-designed devices [2–4], have been used successfully to transfect a wide range of tissues and organisms, including whole animals and plants, bacteria, yeast, mammalian cell lines and even organelles [5–9]. This technique has been particularly successful for biological samples that have previously been difficult or impossible to transfect, such as non-dividing cells or primary neurons [10–12]. Transfection using a gene gun (often referred to as biolistics) is a simple procedure requiring minimal training; it basically consists of two steps: (i) coating microprojectiles with DNA and (ii) firing the coated microprojectiles into the sample.

**Figure 2:**
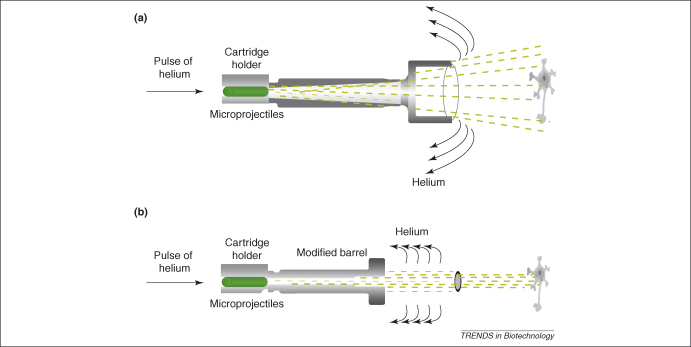
Gene gun-operating principles**.** Example of a gene gun [e.g. The Helios (Bio-Rad) gene gun shown in Figure 1] that uses helium to accelerate microparticles to velocities sufficient to penetrate cells. For this gun, there are two alternative barrels: **(a)** the original and **(b)** the modified version. In the original version, helium gas (usually 100–160 psi) is pulsed through the ‘bullet’, which is a tube loaded with dye-coated microprojectiles that is placed in the cartridge holder. The microprojectiles are swept down the accelerating channel by the helium and, as they leave the cone barrel, they follow the contours of the outer surface, projecting particles over a relatively wide area. This area depends both on the shape of the barrel and on the distance of the gun from the sample. In the modified version, the accelerator channel and its distal cone are replaced by a modified accelerator channel and external barrel, the walls of which are parallel. This barrel has a series of baffle holes that disperse the energy of the helium wave. The result of this is that the area of dispersal of the particles is reduced considerably and the gas pressure required is less (routinely 50–80 psi), resulting in less tissue damage [13].

**Figure 3:**
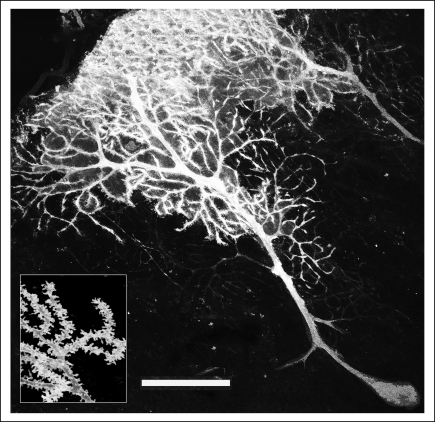
Diolistically labelled cells show considerable detail. A mouse Purkinje cell labelled diolistically using the lipid-soluble fluorescent dye 3,3 dioctadecyloxacarbocyanine perchlorate (DiO) after applying the dye with the modified gene gun is shown. The insert shows a higher magnification of a part of the same image, which enables visualization of spines on dendritic shafts. Examination of these spines revealed that they were arranged in a helical fashion [14]. (Scale bar = 50 μm).

Perhaps unsurprisingly, because the methods for introducing nucleic acids into cells have been so successful, biolistics is now being used to introduce dyes into cells in a technique that has become known as diolistics. Here, dyes are coated onto microprojectiles or filters and are then ‘fired’ into the sample tissue as described earlier. The combination of non-toxic dyes, such as the carbocyanine dyes (Box 1), and highly efficient transportation into cells has permitted labelling of many cell types in their entirety in living tissue over relatively long periods. Modifications made to the Helios gene gun have been particularly useful here (Figure 2) because they enhance the accuracy of the gun by restricting its target area and they also increase the depth penetration achieved by the microprojectiles, enabling deeper tissues to be transfected [13]. An example of the success of this method is the 3D imaging of spines on cerebellar Purkinje cells (Figure 3), which has shown, for the first time, that they are arranged in a helical fashion [14].

## Theoretical considerations

The gene gun achieves successful transportation of DNA and dyes into cells by propelling appropriately coated heavy metal microprojectiles at high velocity so that they penetrate cell walls and membranes. Thus, important considerations are the design of the gun, in particular the mechanism and velocity of the propulsion, and the production of the coated microprojectiles or ‘bullets’. The first gene guns used gunpowder to propel the microprojectiles, however, the current generation usually use a high pressure gas wave or a high-voltage electric spark. An example of how a gas-driven gun (the Helios gene gun) works is shown in Figure 2a. Here, the microparticles are propelled by helium, which is inert and readily dispersible and therefore often the gas of choice. The pressure used depends on the characteristics of both the gun and the tissue sample and needs to be determined experimentally for each novel combination, taking into account the results required; higher pressure ensures more microprojectiles reach their target but also causes more cell damage. The size of the microprojectiles is also important [15] and, indeed, a mathematical model of the process has concluded that microprojectile size has a greater effect on the penetration depth than the helium pressure [16]. Using a modified barrel on this gun (Figure 2b) enables helium to escape before it hits the target, which results in less tissue damage [13]. The design also enables the microprojectiles to be more focused, resulting in deeper penetration. This modification is, therefore, especially useful for the efficient transfection of deep tissues.

The microprojectiles can, in theory, be constructed from any inert heavy metal; most studies have used gold or tungsten and there are also reports of silver microprojectiles [3]. Crucial factors here are that the particles are non-toxic and can be readily coated with the substance being transported; uniformly sized particles can also assist accurate and reproducible labelling. For DNA transfections, gold is often preferred because the DNA on tungsten particles might be more susceptible to catalytic degradation than the DNA on gold particles and there are some reports of tungsten particles acidifying culture medium [1]. Dyes are usually dried directly onto microparticles after solvation in an appropriate solvent or water. The microparticles are then placed onto a filter support or into tubes to create a bullet (see [17] for more details). Bullets are stable for many weeks if stored appropriately.

## Advantages of diolistic labelling

Microinjection has been the method of choice for many years for incorporating fluorescent dyes into cells. Dyes, such as Lucifer Yellow and carboxyfluorescein, have proved to be popular fluorescent compounds for determining cellular architecture. Microinjection, however, is technically demanding and time consuming, and only a small number of cells can be labelled at any one time. In addition, loading cells this way might dialyze the cellular contents and, thereby, might disrupt the concentration of vital cellular components, so effecting cell function(s). There is also a limitation to the cell types that can be used readily for microinjection. Cultures that grow in suspension are obviously difficult to use, as are small adherent cells, and it is not possible to label cells that are several layers deep into tissues.

The diolistic approach has several advantages over microinjection: The technique is rapid and simple to perform; many cells can be labelled simultaneously; and, because the microparticles are small and inert, there is no disruption to cellular processes. Many types of preparation are suitable because even very small cells can be labelled, although to date only adherent cells or whole tissues have been used.

Other routes that have been used to insert dyes into cells include: electroporation, which can be efficient but requires cells in suspension and is dependent on cell type and specific conditions; and lipofection with commercially available lipid transfection agents, which can be simple and efficient but might be toxic to cells, has some limitations in what it can deliver and can be expensive [18,19]. Other potential methods include sonoporation, which uses ultrasound to increase cell permeability, or the use of cell-penetrating peptides or chemicals, such as cytochalasin D, which form membrane pores [18,19]. There are too few studies currently using these methods to critically evaluate them but studies so far indicate that they are unlikely to prove superior to using the gene gun.

In addition to circumventing the problems described here, there are two other advantages of using diolistics with lipophilic dyes to study cell morphology. First is speed because labelling of cells with the carbocyanine dyes is usually complete within minutes. Such rapid labelling enables the investigation of tissue that deteriorates rapidly post-mortem, such as brain tissue. In brain slice experiments investigating neuronal processes, for example, the health of the preparation can be maintained for only 2–3 h at room temperature [20,21]. Here, the use of the carbocyanine dyes compares favourably with the use of green-fluorescent protein, for example, which requires many hours for gene expression. Second, the labelling technique involves only passive dye transfer and diffusion that is independent of gene transcription and protein synthesis, thus, all types of cells can be labelled non-selectively in both fixed and living tissue.

## Current uses of diolistics

A range of different dyes has been delivered using diolistics, including the carbocyanine dyes (Box 1), many ‘classic’ fluorescent dyes, voltage-sensitive dyes, dextran-conjugated pH indicators and ion-selective dyes; these different dyes have been applied to explore a wide range of scientific questions. Diolistics has proved to be a simple and rapid route to examine cell architecture and morphology and, because it is non-invasive and can be used in living tissue, it can also probe complex situations. Studies, for example, have used the diolistic approach with a rhodamine-tagged tracer to show specific migration patterns of cells in embryonic brains [22] and a range of gap junction-permeably dyes (including carboxyfluorescein, Lucifer Yellow and Alexa 488) have been used to determine that the patterns of cell–cell coupling in embryonic spinal cord can be highly dynamic [23].

The information from ion- and pH-sensitive dyes has the potential to be revolutionized by the use of diolistics. Fluorescent calcium-sensitive dyes, for example, delivered diolistically into neuronal tissue have provided the first direct evidence that local calcium release can regulate dendritic structure [24,25]. A particular advantage here is in the ability to deliver these dyes to all types of cells; calcium-sensitive dyes can be loaded readily into immature tissue or cultured cells by incubating tissues with membrane-permanent forms, such as acetoxymethyl (AM) esters, however, this is not effective for many preparations [26,27]. Calcium-sensitive dyes have also been introduced into plant cells using diolistics, including guard cells of *Commelina communis*, cells of the green alga *Chlamydomonas reinhardtii* and zygotes of the brown alga *Fucus serratus,* and this has revealed some novel oscillations in their intracellular calcium concentrations [28].

## Nanosensors

One of the potentially most useful applications of this technique is in the transportation of optical nanosensors. A nanosensor is a device that is less than 1000 nm in diameter and that can ‘sense’ and ‘report’ a chemical or biological event. Nanosensors are therefore ideal for monitoring events within tissues or cells or even within some intercellular organelles because they can be located close to the site of action yet cause minimal physical perturbation. Optical nanosensors are those that report using an optical signal, such as fluorescence [29]. Many such nanosensors have been developed in the laboratory of Kopelman, who refers to them as PEBBLEs (photonic explorers for bioanalysis with biologically localized embedding) [30]. PEBBLEs, and indeed other types of nanosensor, face the conundrum of needing their sensing mechanisms to be accessible to the immediate environment, whereas the reporting mechanism must be protected from interference from this environment. There are a variety of mechanisms used to overcome these problems; the first reported optical nanosensor, which was used for pH sensing, used a fluorescent probe entrapped in a polyacrylamide nanoparticle [31]. Since then, polyacrylamide-based nanosensors have been developed for a range of substances and there has also been increasing use of another matrix, silica sol-gel. This chemically inert, optically transparent material can be processed to define specific properties of the matrix (e.g. pore size and hydrophobicity) such that these are optimised for the sensing and reporting molecules. Currently, nanosensors have been developed to detect a range of substances, including calcium, zinc, magnesium, glucose and even organophosphate insecticides [30,32–35].

Accurate incorporation of the appropriate number of optical nanosensors into the appropriate cells is crucial to obtain an appropriate fluorescent signal. There are a range of possible transportation methods, including cell-penetrating peptides, pinocytosis, lipid transfection agents and picoinjection (see [19] for review), however, the use of the gene gun has proved to be both efficient and effective and is thus considered by many to be the method of choice. A single ‘shot’ can deliver many sensors that are spread evenly throughout the cell and, once the correct conditions have been determined (e.g. pressure and nanosensor concentration), cell viability can be as high as 98% [17,33].

Thus, the rapid and continuing advance in the development of different nanosensors, combined with an effective and accurate method if inserting them into cells, will enable much progress in this field in the future.

## Conclusions

Diolistics has many advantages compared with other techniques of incorporating dyes into cells and we envisage that it will have a major role in improving our understanding of the structure and function of cells and parts of cells that have proved difficult to study previously. The modifications to the Helios gene gun have shown that lipophilic dyes can penetrate up to 500 μm [13] providing the potential to study areas deep within tissues. The development of the capillary-type gene gun has shown that it is possible to target the microprojectiles to very discrete areas (e.g. [4]) enabling, for example, the monitoring of ion or pH changes in individual cells. Combining this technique with advances in microscopy has the potential for extremely complex studies, for example, the use of a miniaturized two-photon microscope, which has enabled fluorescence imaging with subcellular resolution in the brain of awake, freely moving animals [36–38], should enable specific biological events deep in tissues to be examined in living animals. Further development of nanosensors, combined with the use of the gene gun to deliver them, will enable the monitoring and quantification of an even greater range of cell metabolites in a range of cells and tissues. We look forward with anticipation to many potential advances.

## Figures and Tables

**Figure I:**
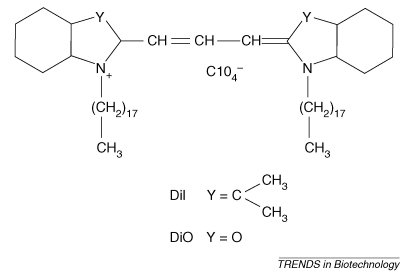
Structure of carbocyanine dyes. For Dil Y = C(CH_3_)_2_ and for DiO Y = O.

## Box 1 Carbocyanine dyes

A wide variety of fluorescent molecules have been used to label cells. Some, such as Lucifer Yellow, have been available for many years, whereas others, such as the range of Alexa Fluor dyes, have only been developed recently. A wide range of characteristics (e.g. cellular architecture, enzyme activity, ion concentrations) can be probed with such dyes (see current uses of diolistics section) and, thus, it is not surprising that this is a very active area of development.

One group of dyes that has evoked much interest recently is the carbocyanine dyes. These dyes are a family of intensely fluorescent molecules; they are weakly fluorescent in water but highly fluorescent and photostable when incorporated into membranes [39]. The structure of the carbocyanine dye is essentially two positive-charged conjugated rings attached to a long hydrocarbon chain by methine groups. The hydrocarbon chains embed into the lipid bilayer between the phospholipid acyl chains, with their conjugated bridge chromophore parallel to the surface of the cell. Related dyes have minor alterations in their ring structure and it is the particular ring structure and number of methine groups that determine the fluorescence characteristics of the molecule. The most widely used dyes for biolistic delivery at present are DiO (3,3 dioctadecyloxacarbocyanine perchlorate) and Dil (1,1-dioctadecyl-3, 3,3,3tetramethylindocarbocyanine perchlorate), which both have an 18 C hydrocarbon chain. Their basic structure is shown in Figure I.

Incorporation of the dye into the membrane of a cell does not seem to interfere with growth or other physiological properties and the dyes diffuse with the mobility of native lipids (≤6 mm per day) [39]. Thus, even when the dye is applied to only a portion of a cell (such as an axon or cell body of a nerve cell), the entire cell surface can become brightly labelled very rapidly. This has permitted the detailed examination of structures of individual cells in living tissue and the investigation of interactions among different types of cells, for example, the dynamic aspects of neuronal connections [40,41]. Because these dyes spread by diffusion rather than by active transport, they are reliable tracers as long as the cell membranes remain intact. This also means that they can be used to label cells in fixed tissue, although dye mobility here is lower and only 0.2–0.6 mm per day [39]. This increases their potential usefulness; for example, it should be possible to map neuronal projections in the human brain using post-mortem tissue [42,43].
